# Electrical Properties and Performance Enhancement of AlGaN/GaN/Si HEMTs

**DOI:** 10.3390/mi17030297

**Published:** 2026-02-27

**Authors:** Hana Mosbahi, Mohammed Khalil Mohammed Ali, Malek Gassoumi

**Affiliations:** 1 Laboratoire de Micro-Optoélectroniques et Nanostructures (LMON), Département de Physique, Faculté des Sciences de Monastir, Université de Monastir, Monastir 5019, Tunisia; 2 Department of Physics, College of Science, Imam Mohammad Ibn Saud Islamic University (IMSIU), Riyadh 11623, Saudi Arabia; 3 Laboratory of Condensed Matter and Nanoscience, Department of Physics, University of Monastir, Monastir 5019, Tunisia

**Keywords:** AlGaN/GaN/Si HEMTs, DC measurements, pulsed current voltage, small-signal microwave, electron trap, DLTS

## Abstract

This study presents a detailed electrical analysis of AlGaN/GaN/Si HEMTs grown by molecular beam epitaxy, using direct and pulse current, small-signal microwave, and deep-level transient spectroscopy (DLTS) techniques to investigate transport characteristics and defect-related effects. DC measurements revealed self-heating effects and leakage currents, while RF analysis highlighted the devices’ high-frequency capabilities alongside parasitic effects linked to deep-level traps. Pulsed I–V characterization demonstrated gate-lag and drain-lag behaviors associated with dynamic charge trapping. DLTS identified electron traps, emphasizing their critical role in device degradation and switching performance. The strong correlation between trap states and electrical behavior underlines the importance of defect control for enhancing efficiency and reliability.

## 1. Introduction

Gallium nitride (GaN)-based high electron mobility transistors (HEMTs) have attracted considerable attention for high-power and high-frequency electronic applications owing to the intrinsic physical properties of GaN, including its wide bandgap, high breakdown electric field, high electron saturation velocity, and excellent thermal stability. These advantages enable GaN-based devices to outperform conventional silicon technologies in demanding operating conditions, particularly in power amplification, microwave systems, and energy conversion applications.

Among the various GaN device configurations, AlGaN/GaN HEMTs grown on silicon substrates represent a highly attractive solution due to the combination of superior electrical performance and reduced fabrication cost [1,2]. The use of large-diameter silicon wafers enables scalable manufacturing and compatibility with existing CMOS infrastructure, which is essential for industrial deployment. As a result, AlGaN/GaN/Si HEMTs have emerged as promising candidates for next-generation power electronics, radio-frequency communication systems, and transportation-related technologies requiring high efficiency and reliability [3,4,5].

Despite significant progress in device design and material growth, the performance and long-term stability of AlGaN/GaN/Si HEMTs are still limited by various degradation mechanisms. In particular, the presence of electrically active defects and trap states in the barrier, buffer, and heterointerface regions can induce current collapse, gate-lag and drain-lag effects, and reduced switching speed. These phenomena are strongly influenced by charge trapping and de-trapping processes, which alter the two-dimensional electron gas (2DEG) density and degrade both static and dynamic device characteristics [6,7,8,9]. Furthermore, self-heating effects under high bias conditions can exacerbate these issues by reducing carrier mobility and accelerating reliability degradation.

A comprehensive understanding of carrier transport and defect-related phenomena is therefore essential to optimize the electrical performance and operational stability of AlGaN/GaN/Si HEMTs. Combining direct current (DC), radio-frequency (RF), pulsed current–voltage, and defect spectroscopy techniques provides a powerful approach to correlating macroscopic electrical behavior with microscopic defect states [7,10].

In this work, we present an in-depth electrical characterization of AlGaN/GaN/Si HEMTs using DC, pulsed I–V, small-signal RF measurements, and deep-level transient spectroscopy (DLTS). The objective is to investigate self-heating effects, dynamic trapping phenomena, and the role of deep-level defects in device performance degradation. By establishing correlations between transport characteristics and trap-related effects, this study aims to provide valuable insights for improving the efficiency, reliability, and robustness of GaN-based HEMTs for advanced power and RF applications.

## 2. Experimental

Molecular beam epitaxy (MBE) is used to generate the AlGaN/GaN HEMTs under study on a silicon (111) substrate. The active layers include a 1 nm n^+^- GaN cap layer, a 1.8 µm undoped GaN channel, a 23 nm thick undoped Al_0.26_Ga_0.74_N barrier, and a 500 nm thick undoped AlN/AlGaN buffer. E-beam lithography is used to design the ohmic contact pads. The metallization is then deposited at 900 °C for 30 s using evaporated 12/200/40/100 nm Ti/Al/Ni/Au. The Schottky gate is implemented with 100/150 nm Mo/Au layers. In contrast, 100/50 nm SiO_2_/SiN with NH_4_OH pretreatment passivates the AlGaN/GaN HEMTs.

A Hewlett-Packard 4142B (Yokogawa-Hewlett-Packard (YHP), Tokyo, Japan) modular parameter analyzer was used to detect DC measurements. To guarantee constant measurement conditions, all tests were carried out in the dark and at atmospheric pressure. Using a capacitance meter (PAR 410, Algam Lighting, Thouaré Cedex, France) with lock-in detection, electrically active defects in AlGaN/GaN/Si HEMTs were characterized using Deep Level Transient Spectroscopy (DLTS). S-parameters measurement is an important step for estimat-ing RF-performance of power component, and to extract thevarious parameters of linear model. Measurements of theseparameters were performed under coplanar points up to 60GHz using a vector network analyzer (HP 8510, Yokogawa-Hewlett-Packard (YHP), Tokyo, Japan) to characterize samples.

## 3. Direct Current Characteristics

**Figure 1:**
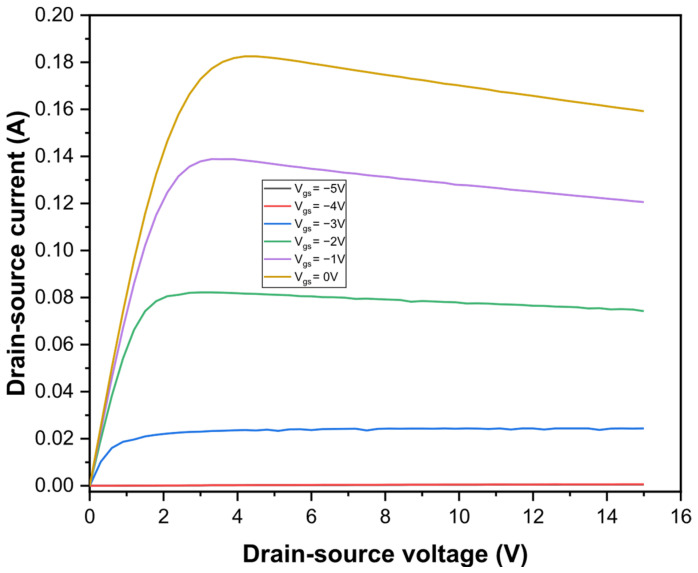
Direct-current characteristics of the AlGaN/GaN/Si HEMT devices at different biases. voltages.

Direct current measurements were conducted on AlGaN/GaN/Si HEMTs at room temperature to analyze their electrical behavior. Figure 1 shows the drain-source current as a function of drain-source voltage at different biases for gate- source voltage. The maximum observed drain-source current reached 0.18 A. A noticeable degradation in direct current performance was observed with increasing drain-source voltage, which can be attributed primarily to the self-heating effect within the device. This self-heating results in an increase in the local temperature of the two-dimensional electron gas (2DEG) channel, causing a reduction in electron mobility. The elevated temperature adversely affects carrier transport, thereby limiting current saturation and degrading device performance [11,12]. Such thermal effects are critical considerations in high-power operation of AlGaN/GaN/Si HEMTs and highlight the importance of efficient thermal management to maintain device reliability and performance [13,14,15,16].

**Figure 2:**
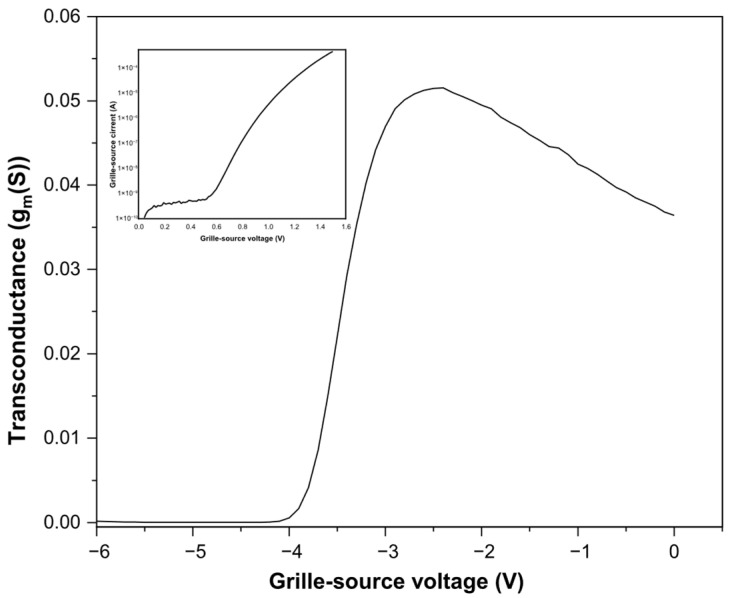
Transconductance and Igs-Vgs characteristics of the AlGaN/GaN/Si HEMT devices.

The transconductance is a key performance indicator that reflects how efficiently the AlGaN/GaN/Si HEMT converts gate-voltage variations into drain-current modulation. Figure 2 shows the measured transconductance, which reaches a value of 0.05 S. This level is mainly influenced by the two-dimensional electron gas density, the carrier mobility within the channel, the AlGaN barrier properties, and thermal effects that may reduce mobility under high bias [17]. Gate-leakage behavior, extracted from the logarithmic Igs–Vgs characteristics shown in the inset of Figure 2, provides insight into the quality of the Schottky contact. A low gate-leakage current indicates a well-formed metal–semiconductor interface, whereas any increase may signal defects or barrier inhomogeneities affecting device reliability. The electron transport parameters are summarized in Table 1. The static parameters of the AlGaN/GaN/Si HEMTs transistors, such as transconductance, leakage current, barrier height, and ideality factor at Vgs = 0 V, were determined. These results provide a reference to emphasize the role of material properties and interface quality on carrier transport and leakage behavior. It should be noted that the leakage current is due to surface defects or finite barrier height [18,19].

## 4. Radio-Frequency and Pulsed Measurements

RF measurements for AlGaN/GaN HEMTs are essential for characterizing, optimizing, and ensuring reliable device performance. These measurements provide critical insights into the capabilities and limitations of AlGaN/GaN HEMTs [20]. The current gain (h_21_), power gain (U), current gain and maximum power gain cutoff frequencies (f_t_, f_max_) have deduced from the S-parameters. The values are reported in Table 2. It is found that the values are mainly attributed to the influence of thermal effects at the AlGaN/GaN interface. Literature studies [21] indicate that self-heating and parasitic effects can degrade gain and cutoff frequencies, highlighting the need for optimized barrier thickness, gate design, and thermal management. Correlating these RF metrics with device structure allows identification of strategies to enhance both performance and long-term stability. Additionally, monitoring RF behavior under operational stress provides an indirect assessment of reliability, as deviations can indicate early degradation or trap-related effects. This integrated approach ensures that AlGaN/GaN HEMTs can achieve high efficiency while maintaining robust operational reliability.

**Figure 3:**
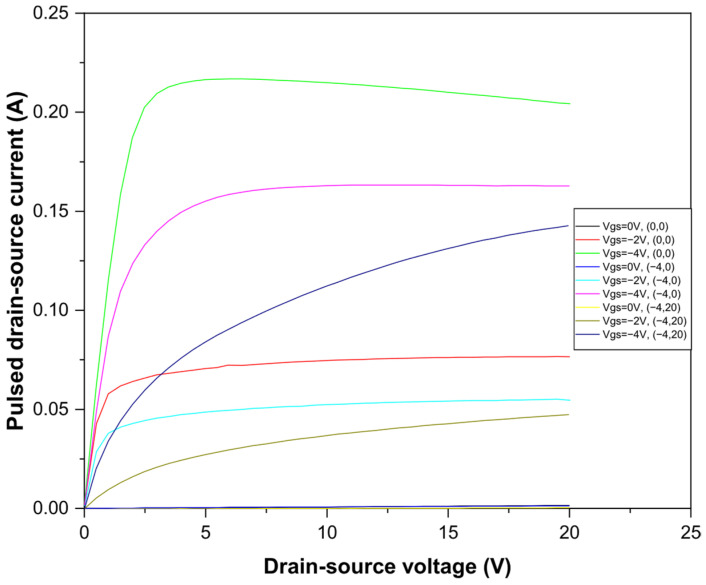
Pulsed Ids-Vds characteristics of the AlGaN/GaN/Si HEMT transistors for three different quiescent bias points.

Pulsed measurements are a powerful tool for identifying and analyzing gate-lag and drain-lag effects in AlGaN/GaN HEMTs. They provide critical insights into the transient behaviors and trapping mechanisms that affect device performance. Pulsed measurements were performed on AlGaN/GaN/Si HEMTs with a pulse width of 500 ns and a period of 10 µs [22]. Three quiescent bias points were used to analyze gate-lag and drain-lag effects. Figure 3 shows the pulse characteristics determined at different quiescent biases. A decrease in the drain current was observed, indicating the presence of electron traps near the gate and drain electrodes. Gate-lag is associated with traps at the AlGaN surface, while drain-lag is linked to slower electron traps in the buffer or surface/barrier interface [23]. These observations highlight how trap states influence dynamic performance and suggest that optimization strategies, such as surface passivation and barrier engineering, can mitigate lag effects. Integrating pulsed measurement results into device design not only improves switching speed but also enhances long-term reliability, providing a practical approach to optimizing AlGaN/GaN HEMTs for high-performance applications.

## 5. DLTS Measurements

In order to study the electrical field effect on the thermal ionization energy of defects, we have used the DLTS technique. This technique allows us to determine the apparent ionization energy *E*_i_ of the trap. This is obtained by performing the measurement of the rate window *e_n_* versus temperature. This rate is related to the trap ionization energy (*E*_c_ − *E*_i_) and the electron capture cross-section *σ_n_* by the principle of the detailed balance, where *N*_c_ is the effective density of states in the conduction band, and *v*th is the mean thermal velocity of the carriers. *k*_B_ is the Boltzmann constant.

The slope of the ln(*e_n_*/T^2^) versus T^−1^ gives *E*_i_. Our measurements were performed in the temperature range of about 20–320 K.

In the depletion region, the electric field *F* varies linearly with the distance from the junction interface, where it is maximum. The variation in *E*_i_ versus *F* is obtained by selecting a narrow region of this depletion region in which *F* can be considered as a constant. A given depth being selected from the filling pulse amplitude, the measurements consist of achieving the difference between capacitance transients monitored for two pulses of slightly different amplitudes, while keeping the reverse bias constant.

DLTS spectra were recorded at constant reverse bias (*V*_0_ = −3 V) with increasing values of the filling pulses Δ*V* and for various values of the rate window *e_n_.*

In the DLTS measurements, negative filling pulses were applied to modulate the depletion region width, with the maximum pulse amplitude limited to 0 V. The reverse bias (*V*_0_) was systematically varied to evaluate its influence on the DLTS signal.

Among the tested conditions, only the spectra recorded at *V*_0_ = −3 V exhibited a stable signal-to-noise ratio and reproducible transient responses, allowing reliable extraction of the trap parameters (activation energy and capture cross-section). For other reverse bias values, the DLTS signal was dominated by significant noise and unstable capacitance transients, which prevented accurate analysis and led to non-reproducible results.

Therefore, only the spectra corresponding to *V*_0_ = −3 V were included in the manuscript, as they represent the only measurement condition providing physically meaningful and quantitatively reliable information.

**Figure 4:**
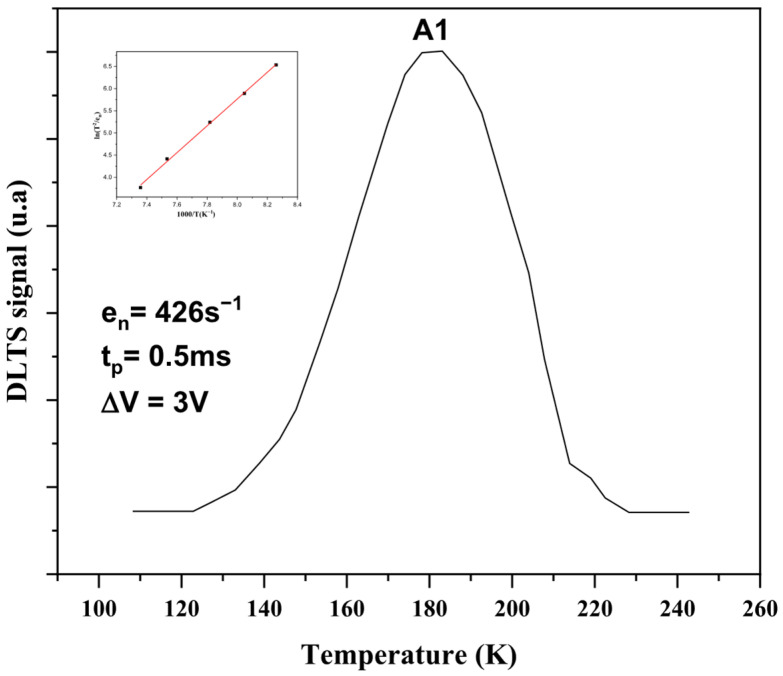
DLTS spectra of the AlGaN/GaN/Si HEMTs. The inset illustrates the binding energies of Traps.

Figure 4 shows the DLTS signal of AlGaN/GaN/Si HEMT. It is found that the DLTS signal is composed of one peak. The deep electron trap is named A1. The binding energies of the trap are evaluated from their signatures. The energy level and the capture cross section are deduced from the Arrhenius diagram of ln($\frac{T^{2}}{e_{n}}$) versus $\frac{1000}{T}$. Signatures of these defects are determined from the Arrhenius plot, as illustrated in the inset of Figure 4. The deep center A1 is characterized by an ionization energy of 0.24 eV and a capture cross section of 6.28 × 10^−16^ cm^2^. The detected trap closely resembles the one previously identified by Mosbahi et al. [24,25,26] in MBE-grown AlGaN/GaN/Si structures using DLTS and CDLTS techniques. A similar defect was also reported by Fang et al. [27] in HVPE-grown n-type GaN. It is suggested that the electron trap A1 may result from a complex involving a pair of N- and Ga- vacancies and is likely located at the AlGaN/GaN heterointerface, playing a significant role in device performance and reliability.

## 6. Discussion

The experimental results presented in this work provide consistent evidence that both transport properties and defect-related phenomena play a critical role in determining the performance of AlGaN/GaN/Si HEMTs. DC measurements revealed a gradual degradation of the drain current at high drain-source voltages, which can be primarily attributed to self-heating effects. The increase in channel temperature leads to reduced electron mobility in the two-dimensional electron gas, thereby limiting current saturation and highlighting the importance of thermal considerations for high-power operation.

The transconductance characteristics reflect the combined influence of carrier density, mobility, and AlGaN barrier properties. While the measured gate leakage current indicates reasonably good Schottky contact quality, the presence of leakage paths suggests that surface states and barrier non-uniformities remain active and may contribute to performance variability. These static electrical parameters provide a baseline for understanding the dynamic behavior observed under pulsed and RF excitation.

Small-signal RF measurements demonstrate that the cutoff frequency and maximum oscillation frequency are sensitive to both intrinsic device parameters and extrinsic effects such as parasitic resistances and self-heating. The observed limitations in RF performance are consistent with thermally induced mobility degradation and trap-related effects, which reduce transconductance and increase access resistance. These findings underline the need for optimized device geometry and improved thermal and material engineering to achieve stable high-frequency operation.

Pulsed I–V characterization revealed pronounced gate-lag and drain-lag effects, confirming the presence of charge trapping phenomena that dynamically affect channel conductivity. Gate-lag behavior is mainly associated with fast traps located at the AlGaN surface or near the gate region, whereas drain-lag effects are attributed to slower traps situated in the buffer layer or at the AlGaN/GaN heterointerface. These trapping mechanisms lead to temporary channel depletion during switching, resulting in reduced drain current under dynamic conditions.

The DLTS measurements support these observations by identifying a dominant deep electron trap with an activation energy of approximately 0.24 eV. The characteristics of this defect suggest a vacancy-related origin near the heterointerface, consistent with previously reported studies on GaN-based heterostructures. This deep-level trap plays a key role in charge capture and emission processes, thereby directly influencing both dynamic performance and long-term device reliability.

Overall, the strong correlation between DC, RF, pulsed I–V, and DLTS results demonstrates that trap-induced effects, combined with self-heating, are major limiting factors in AlGaN/GaN/Si HEMTs. Addressing these issues requires targeted strategies such as improved surface passivation, optimized buffer design, and defect control during epitaxial growth.

## 7. Conclusions

In this work, a comprehensive electrical investigation of AlGaN/GaN/Si HEMTs has been carried out using DC, small-signal RF, pulsed I–V, and DLTS techniques. The combined analysis provides a coherent understanding of both steady-state and dynamic device behavior, highlighting the interplay between carrier transport, self-heating effects, and defect-related phenomena.

DC and RF measurements revealed that self-heating significantly impacts carrier mobility and limits both current drive capability and high-frequency performance. Pulsed I–V characterization demonstrated the presence of gate-lag and drain-lag effects, confirming that charge trapping mechanisms dynamically modulate channel conductivity during switching operation. DLTS analysis identified a dominant deep electron trap located near the AlGaN/GaN heterointerface, which plays a critical role in trapping and detrapping processes and contributes to performance degradation and reliability concerns.

The strong consistency between transport measurements and defect spectroscopy emphasizes that electrically active traps are a key limiting factor in AlGaN/GaN/Si HEMTs. Improving material quality, surface passivation, and buffer engineering is therefore essential to mitigate trapping effects and enhance device stability. The results of this study provide valuable insights for optimizing GaN-based HEMTs and support the development of reliable, high-efficiency power and RF electronic systems.

## Figures and Tables

**Table 1:** AlGaN/GaN/Si HEMT electron transport parameters.

I_ds_ (A)	g_m_ (mS/mm)	Igs0	Vth (V)	η	V_b_ (V)	R_s_ + R_i_ (Ω)
0.18	188	4.03 × 10^−12^	−3.8	1.7	0.90	3.2

**Table 2:** RF parameters acquired for AlGaN/GaN/Si HEMT devices.

h_21_ (dB)	f_t_ (GHz)	f_max_ (GHz)	F_MSG_ (GHz)
9.8	40	56	52

## Data Availability

The original contributions presented in this study are included in the article. Further inquiries can be directed to the corresponding author.
